# Interferon gamma release assay and sputum GeneXpert positivity for tuberculosis burden detection in people deprived of liberty in Brazil: a cross-sectional study

**DOI:** 10.1186/s12889-026-27643-3

**Published:** 2026-05-12

**Authors:** Ghislaine Gonçalez de Araujo Arcanjo, José Victor Bortolotto Bampi, Karina Marques Santos, Michele Souza Ventura, Everton Ferreira Lemos, Eduarda Gassen Boeira, Lia Gonçalves Possuelo, Giselle Lima de Freitas, Beatriz do Carmo Veloso de Oliveira, Jaquelane Silva Jesus, Allyson Guimarães Costa, Marcelo Cordeiro-Santos, Mariana Pinheiro Alves Vasconcelos, Mayara Gonçalves Tavares, Rosilene Ruffato, Beatriz Barreto-Duarte, Pedro P. Carneiro, Bruno B. Andrade, Rebecca A. Clark, Katherine A. Thomas, Richard G. White, Julio Croda

**Affiliations:** 1 Faculdade de Medicina, Universidade Federal de Mato Grosso do Sul, Campo Grande, Brazil; 2 Fundação Osvaldo Cruz, Campo Grande, Brazil; 3 Universidade Estadual de Mato Grosso do Sul, Campo Grande, Brazil; 4 Programa de Pós Graduação em Promoção da Saúde, Universidade de Santa Cruz do Sul, Santa Cruz do Sul, Brazil; 5 Departmento de Enfermagem Materno-Infantil e Saúde Pública, Universidade Federal de Minas Gerais, Belo Horizonte, Brazil; 6 Universidade do Estado do Amazonas, Manaus, Brazil; 7 Fundação de Medicina Tropical Dr Heitor Vieira Dourado, Manaus, Brazil; 8 Universidade Federal do Amazonas, Manaus, Brazil; 9 Universidade Nilton Lins, Manaus, Brazil; 10 Centro de Pesquisa em Medicina Tropical de Rondônia (CEPEM), Porto Velho, Brazil; 11 Institute for Research in Priority Populations (IRPP), MONSTER Institute, Salvador, Brazil; 12 Laboratory of Clinical and Translational Research, Gonçalo Moniz Institute, Oswaldo Cruz Foundation (Fiocruz), Salvador, Brazil; 13 Instituto de Pesquisa Clínica e Translacional (IPCT), Faculdade Zarns, Clariens Educação, Salvador, Brazil; 14 Department of International Health, Bloomberg School of Public Health, Johns Hopkins University, Baltimore, USA; 15 Division of Infectious Diseases, Department of Medicine, Johns Hopkins University, Baltimore, USA; 16 Tuberculosis and Vaccine Centre, London School of Hygiene and Tropical Medicine, London, UK; 17 Department of Epidemiology of Microbial Diseases, Yale University School of Public Health, New Haven, United States

**Keywords:** Tuberculosis prevalence, *Mycobacterium tuberculosis* infection, people deprived of liberty, Brazil

## Abstract

**Background:**

Globally tuberculosis disease (TB) poses a significant public health challenge. People deprived of liberty (PDL) are one of the most affected. This study aims to evaluate the positivity of interferon gamma release assay (IGRA) and molecular sputum testing for TB through a multicenter survey of Brazilian prisons and evaluate factors associated with IGRA positivity.

**Methods:**

We performed a cross-sectional study among PDL in six male and two female prisons within 6 cities in Brazil in 2025. Eligible participants included consenting adults over 18 years. We randomized 130 individuals per site using prison census for evaluation with a sociodemographic questionnaire, blood testing with QuantiFERON-TB Gold Plus^®^ (IGRA) and sputum testing with GeneXpert Ultra^®^ (Xpert). We included individuals with complete data and valid IGRA results for analysis. Multivariable logistical regression was performed to evaluate factors associated with IGRA positivity stratified by gender.

**Results:**

Among 1040 PDL randomized, 884 were included. The overall IGRA positivity was 53.6% (95% confidence interval [CI] 50.3–56.9) varying between 29.2% (21.6–38.2) and 84.0% (75.3–90.1) among prisons sites, male and female individuals had a positivity of 61.9% (58.1–65.5) and 30.3% (24.7–36.5), respectively. In the multivariable analysis, IGRA positivity was associated with lower education (adjusted odds ratio [aOR] 1.46, 95% CI 1.03–2.07, *p* = 0.034), previous incarceration (aOR 2.81, 1.85–4.25, *p* < 0.001), previous TB history (aOR 2.99, 1.39–6.42, *p* < 0.001) and incarceration time (< 1 year as reference, 3–4 years: aOR 2.15, 1.18–3.92, *p* = 0.012; 5 years or above: aOR 2.85, 1.66–4.90, *p* < 0.001) for males. In females, IGRA positivity was associated with previous TB history (aOR 5.93, 1.97–20.2, *p* = 0.002). One prison could not collect sputum samples; at the remaining sites we detected 20 individuals (2.5%, 20/770) with positive Xpert.

**Conclusion:**

Despite variations among prisons and gender, IGRA and Xpert positivity were elevated across sites evaluated in Brazil. These findings underscore the urgent need for systematic and comprehensive tuberculosis screening, prevention, and control strategies in prisons, including the potential evaluation of new preventative technologies such as new TB vaccines.

**Supplementary Information:**

The online version contains supplementary material available at 10.1186/s12889-026-27643-3.

## Background

Tuberculosis (TB) remains as one of the greatest public health challenges globally, affecting over 10 million people each year [1]. With increasing efforts being made to curb TB incidence and mortality with the End TB goals, a better understanding of the current epidemiological situation is needed to plan healthcare responses and guide future research strategies [2]. Targeted evaluations of the prevalence of *Mycobacterium tuberculosis* (MTB) infection and active TB disease performed through active case finding or surveys in high burden groups can provide vital information for policymakers, researchers and public health authorities [3].

People deprived of liberty (PDL) are one of the groups with the highest burden of TB disease worldwide. Previous estimates found that in South America, the TB incidence rate is 27-fold higher in prisons than in the overall population [4]. In Brazil, despite only 0.4% of the population being incarcerated, 9.1% of the new TB cases occurred in PDL in 2023 [5]. These disparities result in carceral settings becoming reservoirs of disease to the surrounding communities and a large excess burden of infection in the national population [6, 7].

Recommendations from the World Health Organization and the Brazilian Ministry of Health suggest that active TB screening should be performed periodically in prisons [8, 9]. However, there is no current systematic screening program in Brazilian prisons. Several groups have performed active case finding studies independently with different methodologies [10–15] but no prevalence estimate was performed consistently across different regions and current data corresponds mostly to passive diagnosis, which may lead to gaps in case detection rates [16].

New surveys can provide updated information about the active TB cases and MTB infection for public health planning, infection control and dynamic infectious diseases modelling. Despite the current understanding that tests evaluating immunoreactivity to MTB are not a perfect measure of the true underlying infection proportion, those results could provide an essential panorama for planning new technologies for disease control [17]. Currently there are several new TB vaccines at the development pipeline, the participation of people deprived of liberty in these trials has been discussed in the context of possible benefits for disease prevention in this vulnerable group and increased efficiency of trials due to the frequency of disease endpoints [18]. Previous studies have assessed that these trials proposals are well accepted by the prison population [19, 20].

Analyzing the data generated from a multicenter cross-sectional survey in prisons across all five Brazilian regions, this paper aims to understand the prevalence of IGRA positivity and active TB disease, evaluating associated sociodemographic and clinic characteristics associated with IGRA positivity.

## Methods

### Study setting and design

We performed a cross-sectional study in six male prisons (Belo Horizonte, Campo Grande, Manaus, Porto Velho, Salvador and Venâncio Aires) and two female prisons (Campo Grande and Manaus) located in six cities in Brazil, covering all five country regions. Individuals incarcerated in those units were all adults (over 18 years old) sentenced to a closed system regimen where individuals do not leave the prison during incarceration. The prisons had variable architecture, where most individuals are held in cell blocks constituted of closed cells organized around open wards. Individuals spend less than 4 h a day outside of the cells, with smaller cell blocks dedicated to specific populations such as workers or solitary confinement. Movement between cells and cell blocks is frequent and is determined by prison staff, however we did not had access to reliable measures of individual movements in each prison. These sites were selected for the study because they already had research teams experienced with prison health research.

In 2025, male prison population varied between 606 individuals (Venâncio Aires) and 1593 individuals (Campo Grande) with a mean occupancy rate of 158%, varying between 105% (Salvador) and 360% (Campo Grande). The female prisons had a population of 220 individuals with 111% of occupancy (Manaus) and 311 individuals with 137% occupancy (Campo Grande) [21]. Only two units, both in Campo Grande, currently perform some screening for active TB disease in their population with frequent mass screening campaigns in both prisons with Xpert and chest X-ray performed for all individuals and an ongoing cohort in the male prison evaluating a proportion of the population every four months with chest X-ray and sputum analysis. The remaining units rely on passive diagnosis alone, where individuals with TB symptoms seek care in the prison clinic and are evaluated. Data collection and study procedures occurred between April 2025 and October 2025.

### Data collection

Prison staff provided study workers with a complete unit census with unique prison identification numbers in the week of study procedures that was used to randomize our sample through simple random sampling. We did not perform any stratification accounting for cell block size, crowding or other site characteristics. After randomization, we obtained name, cell block and cell number of the randomized individuals to be approached for interview. Study procedures occurred inside prison grounds, within the prison wards, so it was necessary for prison staff to know who would be approached to open cell blocks for contact with individuals. Prison staff were not present during interview, neither were they informed about willingness to participate. There were no incentives nor reprimands for individuals to engage in the study procedures.

We estimated from previous studies [22] that a minimum sample size of 428 individuals would be required to detect a 50% proportion of IGRA positivity with a precision of ± 5%, accounting for 10% of losses or refusals to participate. We opted for a sample frame where each site would be equally sampled plus 10 extra individuals per site to account for losses and refusals. Thus, we opted for an equal sampling of 120 PDL for each unit, randomizing 130 individuals from each prison unit to account for losses or refusals. We excluded individuals that did not want to participate and those who were illiterate and would not be able to read the informed consent form. Security measures in each unit only allowed a determined number of participants to be seen each day for a limited period of time, so we stopped inclusion when the sample size approached the most possible number of participants considering prison setting limitations.

Those who were able to read the consent form and were capable of providing informed consent were evaluated through a sociodemographic questionnaire, including carceral history, medical history, contact with TB cases in the same cell, current illicit drug use or tobacco smoking and alcohol abuse related disorders. Individuals self-reported their HIV status; we did not perform HIV testing. A version of the questionnaire translated to English is available in the supplement (Supplementary Material 2 – Annex 1). There were no other exclusion criteria for participation. After initial evaluation, individuals underwent venous blood collection for interferon-gamma release assay (IGRA) testing with the QuantiFERON-TB Gold Plus^®^, processed and categorized according to manufacturer instructions. All individuals were asked to provide one individual sputum sample after IGRA for individual testing with GeneXpert Ultra^®^ (Xpert). After data collection and sample processing we excluded patients with missing questionnaire data, missing or indeterminate IGRA results.

### Laboratory procedures and results management

The QuantiFERON-TB Gold Plus^®^ assay was performed according to the manufacturer’s instructions. The same operator was responsible for sample processing across all sites. Blood samples from each participant were incubated at 36–38 °C for 16–24 h for cell stimulation. After incubation, supernatants were harvested, stored at − 80 °C, and subsequently sent to Campo Grande for batch ELISA testing, ensuring operational protocol standardization. Results were considered valid only when accepted by the QFT-Plus software. A positive result was defined as an IFN-γ response ≥ 0.35 IU/mL above the Nil value in either TB1 or TB2, and indeterminate results were defined per manufacturer criteria (Nil > 8.0 IU/mL or Mitogen < 0.5 IU/mL).

Sputum samples were stored at 2–8 °C on site and transferred on the same day to the local laboratory for Xpert processing, reflecting routine prison healthcare clinic conditions. We reported to prison healthcare providers individuals with positive and trace results to be evaluated by medical consult. According to the Brazilian guidelines, all individuals with positive Xpert results are considered cases and trace results are considered as positive in people living with HIV or if there is clinical suspicion of extra-pulmonary disease. Considering we did not collect follow-up information for the TB cases after medical consult; we considered TB cases to be only the individuals with a positive Xpert result at first evaluation. Individual follow-up, decision to treat and care was performed by the healthcare clinic professionals in each site, according to Brazilian TB care guidelines [9].

### Data management and statistical analysis

The study team collected and stored data in an online RedCap^®^ (Vanderbilt University, Tennessee, USA) database hosted by Fiocruz, Mato Grosso do Sul, Brazil. Data analysis was performed utilizing R statistical software [23]. At first, we conducted Chi-square and Fisher tests for categorical variables to determine differences in the response variables among subgroups of different genders and IGRA results, we also evaluated if there was a trend for positivity with increasing incarceration time through the Cochran–Armitage test. Due to significant differences between male and female groups regarding prison characteristics, demographics and carceral history, we performed a separate analysis of factors associated with IGRA positivity in each group. We utilized the Wilson score interval to obtain confidence intervals for the prevalence of IGRA positivity in the overall population and prison and gender subgroups.

Univariate logistic regression analysis was performed on both groups. A collinearity verification was performed calculating the variance inflation factor for the variables included. The multivariable model aimed to evaluate a predictive model for IGRA positivity in the PDL. We performed a sensitivity analysis of the main model where we selected only the population without previous history of TB, since those individuals might have persistent IGRA positive results after treatment.

For male prisons, we included in the multivariable model all variables that had a strong association with IGRA positivity in the univariate analysis (with a p-value < 0.05), in addition to including age as an a priori confounder; we included prison unit as a random effect in the model to account for the variability between units. For the female prisons analysis, due to a smaller sample size with fewer outcomes, we performed a simpler fixed effects model evaluating previous TB history and variables that are related to period of exposure to the prisons setting (current incarceration time and previous incarceration) due to previous data from Brazilian prisons suggesting an strong association of prison history with TB outcomes [11, 14]. Prison units were also accounted for adjustment as fixed effects in the female model.

## Results

**Fig. 1:**
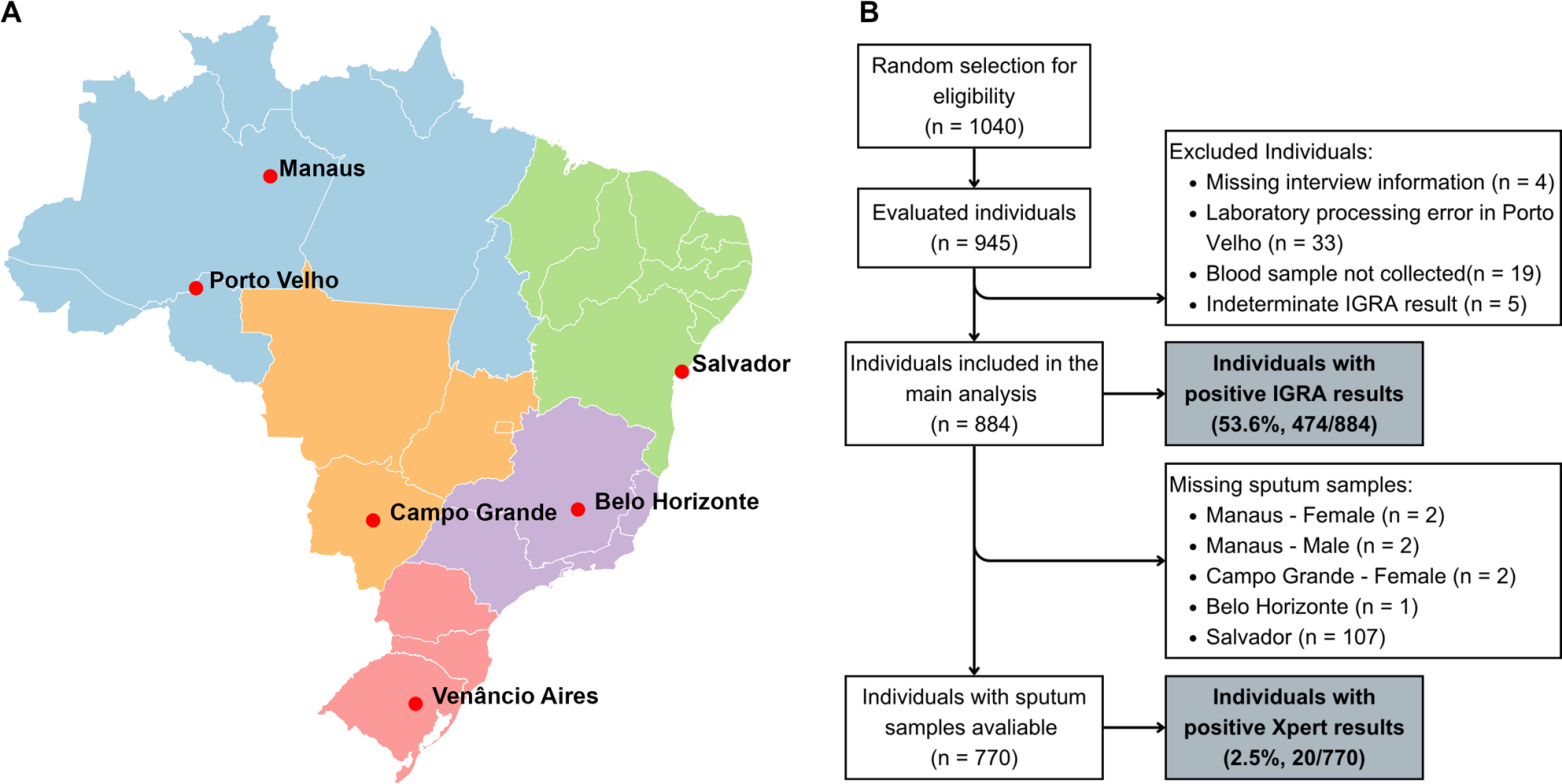
Cities included in the study evaluation and flowchart of inclusion of study participants. Legend: **A** – Cities where the study prison units were located within Brazil. Five diferent country regions are represented in different colors, at least one prison unit was evaluated in each region. **B** – Flowchart of participant inclusion. Abreviations: IGRA: Interferon gamma release assay; Xpert: GeneXpert Ultra

After the first randomization, 1040 individuals were selected for initial approach, 130 individuals in each prison site. In total, 945 individuals were able to be approached and provided informed consent to participate. We excluded four individuals due to missing sociodemographic information, 19 individuals with blood samples not collected, and five individuals with indeterminate IGRA results (two at Campo Grande female prison and three at the Venâncio Aires prison). Due to a lab processing error of samples, 33 individuals from the Porto Velho city prison were also excluded. In the final analysis, 884 individuals were included among the eight prisons (Fig. 1). Sputum samples were not collected for the 107 individuals evaluated Salvador prison and 7 individuals from the remaining sites had missing samples, so the Xpert results are presented for the 770 participants with valid sputum results. The response rate varied between 72.3% (Porto Velho) and 90.7% (Campo Grande – Female), the total number of individuals included for the final analysis and missing sputum samples in each study site is reported in Table 1.

Overall, 26.1% (231/884) of study participants were from female prisons. The evaluated PDL were mostly under 35 years old (61.4%), not white (80.0%) and almost half had incomplete basic education (48.1%). Most individuals had a previous history of incarceration (71.4%) and two thirds of them (66.4%) have been currently incarcerated for up to two years. We encountered significant variation by prison in the proportion of reported history of alcohol abuse (0% to 23.9%), current tobacco smoking (0.9% to 73.5%) and illicit drug use in the last year (2.1% to 65.8%), however the overall proportion of these characteristics was notable (4.6%, 25.8% and 15.5%, respectively). Only 1.5% of individuals reported living with HIV, proportionally similar to the national estimates for the overall population [24]. Among all participants, 8.6% reported previous TB history and 32.9% had previous contact with TB positive individuals in the same cell (Table 1). Individuals differed significantly when compared by gender in most characteristics evaluated, however we found no difference in age, race, previous TB history and HIV status (Supplementary Table 1).

**Table 1:** Characteristics of people deprived of liberty evaluated in a tuberculosis cross-sectional survey, included in the final analysis, by prison unit (*n* = 884)

Variables		Prison City Site							
	**Overall** *N* = 884^*a*^	**MA-F** *N* = 113^*a*^	**MA-M** *N* = 113^*a*^	**CG-F** *N* = 118^*a*^	**CG-M** *N* = 115^*a*^	**PV** *N* = 94^*a*^	**VA** *N* = 117^*a*^	**BH** *N* = 107^*a*^	**SA** *N* = 107^*a*^
Age categories									
18–25 years old	154 (17.4%)	20 (17.7%)	23 (20.4%)	20 (16.9%)	14 (12.2%)	22 (23.4%)	15 (12.8%)	24 (22.4%)	16 (15.0%)
26–35 years old	389 (44.0%)	54 (47.8%)	64 (56.6%)	40 (33.9%)	44 (38.3%)	41 (43.6%)	59 (50.4%)	47 (43.9%)	40 (37.4%)
36–45 years old	238 (26.9%)	22 (19.5%)	18 (15.9%)	42 (35.6%)	40 (34.8%)	26 (27.7%)	32 (27.4%)	28 (26.2%)	30 (28.0%)
Above 46 years old	103 (11.7%)	17 (15.0%)	8 (7.1%)	16 (13.6%)	17 (14.8%)	5 (5.3%)	11 (9.4%)	8 (7.5%)	21 (19.6%)
Race									
White	177 (20.0%)	17 (15.0%)	12 (10.6%)	22 (18.6%)	26 (22.6%)	12 (12.8%)	62 (53.0%)	16 (15.0%)	10 (9.3%)
Black/Mixed and Others	707 (80.0%)	96 (85.0%)	101 (89.4%)	96 (81.4%)	89 (77.4%)	82 (87.2%)	55 (47.0%)	91 (85.0%)	97 (90.7%)
Schooling									
Incomplete basic education	425 (48.1%)	38 (33.6%)	64 (56.6%)	47 (39.8%)	63 (54.8%)	64 (68.1%)	45 (38.5%)	48 (44.9%)	56 (52.3%)
Complete basic education or higher	459 (51.9%)	75 (66.4%)	49 (43.4%)	71 (60.2%)	52 (45.2%)	30 (31.9%)	72 (61.5%)	59 (55.1%)	51 (47.7%)
Previously incarcerated	631 (71.4%)	58 (51.3%)	96 (85.0%)	74 (62.7%)	74 (64.2%)	85 (90.4%)	104 (88.9%)	78 (72.9%)	62 (57.9%)
Previous contact with TB case in the same cell	291 (32.9%)	16 (14.2%)	60 (53.1%)	45 (38.1%)	45 (39.1%)	38 (40.4%)	59 (50.4%)	8 (7.5%)	20 (18.7%)
Current incarceration time									
<1 year	340 (38.5%)	73 (64.6%)	24 (21.2%)	62 (52.5%)	89 (77.4%)	11 (11.7%)	25 (21.4%)	47 (43.9%)	9 (8.4%)
1–2 years	247 (27.9%)	25 (22.1%)	32 (28.3%)	24 (20.3%)	17 (14.8%)	48 (51.1%)	47 (40.2%)	19 (17.8%)	35 (32.7%)
3–4 years	121 (13.7%)	5 (4.4%)	32 (28.3%)	14 (11.9%)	0 (0%)	17 (18.1%)	14 (12.0%)	10 (9.3%)	29 (27.1%)
5 years or above	176 (19.9%)	10 (8.8%)	25 (22.1%)	18 (15.3%)	9 (7.8%)	18 (19.1%)	31 (26.5%)	31 (29.0%)	34 (31.8%)
Previous TB history	76 (8.6%)	7 (6.2%)	9 (8.0%)	8 (6.8%)	13 (11.3%)	13 (13.8%)	19 (16.2%)	1 (0.9%)	6 (5.6%)
Positive IGRA result	474 (53.6%)	33 (29.2%)	78 (69.0%)	37 (31.4%)	49 (42.6%)	79 (84.0%)	74 (63.2%)	60 (56.1%)	64 (59.8%)
Xpert result^3^									
Positive	20/770 (2.5%)	0/111 (0%)	2/111 (1.8%)	2/116 (1.7%)	1/115 (0.8%)	5/94 (5.3%)	7/117(5.9%)	3/106 (2.8%)	**0 (NA)** ^b^
Trace	9/770 (1.1%)	0/111 (0%)	0/111 (0%)	1/116 (0.9%)	0/115 (0%)	1/94 (1.0%)	3/117 (2.5%)	4/106 (3.7%)	**0 (NA)** ^b^
Negative	741/770 (96.4%)	111/111 (100%)	109/111 (98.2%)	113/116 (97.4%)	114/115 (99.2%)	88/94 (93.7%)	107/117 (91.6%)	99/106 (93.5%)	**0 (NA)** ^b^
Missing	114	2	2	2	0	0	0	1	107^b^
Living with HIV	13 (1.5%)	3 (2.7%)	0 (0%)	2 (1.7%)	0 (0%)	0 (0%)	5 (4.3%)	2 (1.9%)	1 (0.9%)
Alcohol abuse	41 (4.6%)	1 (0.9%)	0 (0%)	0 (0%)	2 (1.7%)	0 (0%)	28 (23.9%)	7 (6.5%)	3 (2.8%)
Current tobacco smoking	228 (25.8%)	1 (0.9%)	2 (1.8%)	36 (30.5%)	27 (23.5%)	15 (16.0%)	86 (73.5%)	25 (23.4%)	36 (33.6%)
Current illicit drug use	137 (15.5%)	5 (4.4%)	5 (4.4%)	16 (13.6%)	12 (10.4%)	2 (2.1%)	77 (65.8%)	10 (9.3%)	10 (9.3%)

Abreviations: *MA-F* Manaus – Female, *MA-M* Manaus – Male, *CG – F* Campo Grande – Female, *CG – M* Campo Grande – Male, *PV* Porto Velho, *VA* Venâncio Aires, *BH* Belo Horizonte, *SA* Salvador, *IGRA* Interferon Gamma release assay, *TB* Tuberculosis, *Xpert* GeneXpert Ultra

^*a*^n (%), ^b^Salvador site did not perform sputum testing

**Fig. 2:**
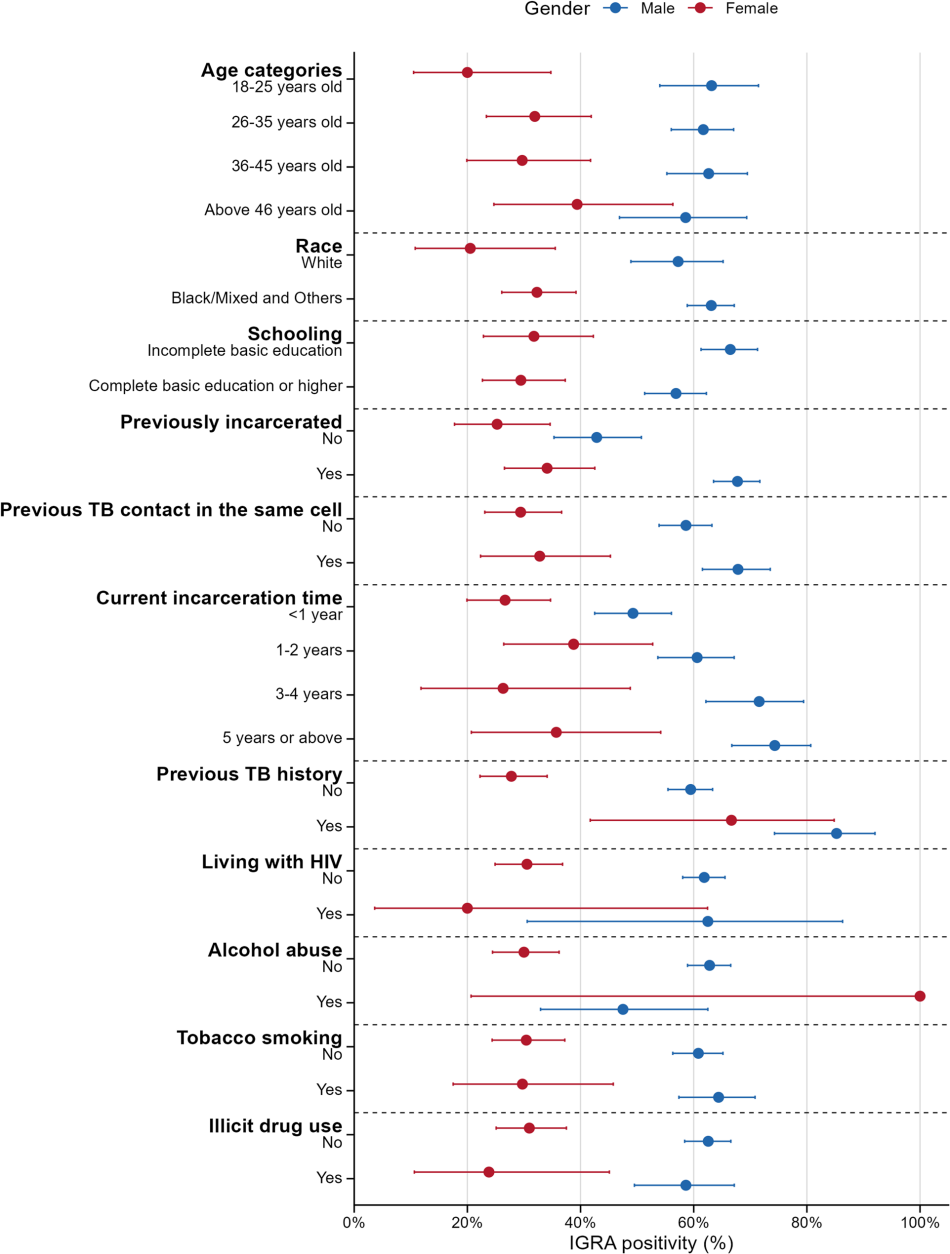
Proportion of IGRA positivity across different subgroups of people deprived of liberty in Brazil, stratified by gender. (*n* = 884). Legend: Values are presented in the measured percentage of positivity with 95% confidence intervals obtained with the Wilson score interval. Small sample sizes for individuals living with HIV (*n* = 13) and females with alcohol abuse history (*n* = 1) distorted the estimates. Abbreviations: IGRA – Interferon gamma release assay; TB – Tuberculosis

The IGRA positivity was high, with an average of 53.6% (95% confidence interval [CI] 50.3–56.9) positivity across all prisons. There was significant variation between sites, the lowest positivity detected was 29.2% (95% CI 21.6–38.2) in the Manaus female prison and the highest was 84.0% (95% CI 75.3–90.1) in the Porto Velho male prison (Table 1). IGRA positivity was considerably higher in male (61.9%, 95% CI 58.1–65.5) than female individuals (30.3%, 95% CI 24.7–36.5, *p* < 0.001) (Supplementary Table 1). A stratified analysis across subgroups also evidenced that IGRA positivity was considerably higher in males then females in most subgroups evaluated (Fig. 2).

Univariate analysis found that in male prisons individuals, a positive IGRA result was associated with lower completion of basic education (44.1% vs. 54.2%, *p* = 0.012), previous TB history (12.9% vs. 3.6%, *p* < 0.001), contact with TB cases in the same cell (38.6% vs. 29.7%, *p* = 0.021) and previous incarceration history (83.7% vs. 64.7%, *p* < 0.001). We also identified an increasing trend in for IGRA positivity with increasing incarceration time (Cochran–Armitage test for trend, *p* < 0.001). In female prisons, a positive IGRA results was associated with previous TB history (14.3% vs. 3.1%, *p* = 0.003) (Table 2). A secondary evaluation for trend stratified by gender and incarceration history identified an increased IGRA positivity by current incarceration time in both males and females without previous incarceration history and males with previous incarceration history (Supplementary Table 2).

**Table 2:** Variables associated with IGRA positivity in people deprived of liberty evaluated in a tuberculosis cross-sectional survey, by gender (*n* = 884)

Variables	IGRA Result - Male			IGRA Result - Female		
	Negative *N* = 249^a^	Positive *N* = 404^a^	*p*-value^b^	Negative *N* = 161^a^	Positive *N* = 70^*a*^	*p*-value^b^
Age categories			> 0.900			0.300
18–25 years old	42 (16.9%)	72 (17.8%)		32 (19.9%)	8 (11.4%)	
26–35 years old	113 (45.4%)	182 (45.0%)		64 (39.8%)	30 (42.9%)	
36–45 years old	65 (26.1%)	109 (27.0%)		45 (28.0%)	19 (27.1%)	
Above 46 years old	29 (11.6%)	41 (10.1%)		20 (12.4%)	13 (18.6%)	
Race			0.200			0.140
White	59 (23.7%)	79 (19.6%)		31 (19.3%)	8 (11.4%)	
Black/Mixed and Others	190 (76.3%)	325 (80.4%)		130 (80.7%)	62 (88.6%)	
Schooling			0.012			0.700
Incomplete basic education	114 (45.8%)	226 (55.9%)		58 (36.0%)	27 (38.6%)	
Complete basic education or higher	135 (54.2%)	178 (44.1%)		103 (64.0%)	43 (61.4%)	
Previously incarcerated	161 (64.7%)	338 (83.7%)	< 0.001	87 (54.0%)	45 (64.3%)	0.150
Previous contact with TB case in the same cell	74 (29.7%)	156 (38.6%)	0.021	41 (25.5%)	20 (28.9%)	0.600
Current incarceration time			< 0.001^c^			0.324^c^
<1 year	104 (41.8%)	101 (25.0%)		99 (61.5%)	36 (51.4%)	
1–2 years	78 (31.3%)	120 (29.7%)		30 (18.6%)	19 (27.1%)	
3–4 years	29 (11.6%)	73 (18.1%)		14 (8.7%)	5 (7.1%)	
5 years or above	38 (15.3%)	110 (27.2%)		18 (11.2%)	10 (14.3%)	
Previous TB history	9 (3.6%)	52 (12.9%)	< 0.001	5 (3.1%)	10 (14.3%)	0.003
Xpert result^d^			0.500			0.400
Positive	3/205 (1.4%)	15/338 (4.4%)		1/158 (0.6%)	1/69 (1.4%)	
Trace	3/205 (1.4%)	5/338 (1.4%)		0/158 (0%)	1/69 (1.4%)	
Negative	199/205 (97.2%)	318/338 (94.2%)		157/158 (99.4%)	67/69 (97.2%)	
Missing	44	66		3	1	
Living with HIV	3 (1.2%)	5 (1.2%)	> 0.900	4 (2.5%)	1 (1.4%)	> 0.900
Alcohol abuse	21 (8.4%)	19 (4.7%)	0.053	0 (0%)	1 (1.4%)	0.300
Current tobacco smoking	68 (27.3%)	123 (30.4%)	0.400	26 (16.1%)	11 (15.7%)	> 0.900
Illicit drug use	48 (19.3%)	68 (16.8%)	0.400	16 (9.9%)	5 (7.1%)	0.500

Abbreviations: *IGRA* Interferon Gamma release assay, *TB* Tuberculosis, *Xpert* GeneXpert Ultra

^a^n (%), ^b^Fisher’s exact test, Pearson’s Chi-squared test, ^c^Cochran-Armitage test for trend, ^d^Salvador site did not perform sputum testing and 7 other sputum samples were missing from the remaining prisons

The multivariable model for male prisons found that IGRA positivity was associated with incomplete basic education (adjusted odds ratio [aOR] 1.46, 95% confidence interval [CI] 1.03–2.07, *p* = 0.034), previous incarceration (aOR 2.81, 95% CI 1.85–4.25, *p* < 0.001), previous TB history (aOR 2.99, 95% CI 1.39–6.42, *p* = 0.005) and longer current incarceration time (< 1 year as reference, 3–4 years: aOR 2.15, 95% CI 1.18–3.92, *p* = 0.012; 5 years or above: aOR 2.85, 95% CI 1.66–4.90, *p* < 0.001). We found no association with age or TB contacts in the same cell for IGRA positivity in the multivariable analysis. The female prison model found that only previous TB history (aOR 5.93, 95% CI 1.97–20.2, *p* = 0.002) was associated with IGRA positivity, with no significant association with previous prison or current incarceration time (Table 3). The sensitivity analysis findings, excluding individuals with previous TB history, were similar to the main model, with no significant change in the coefficients for time of incarceration and previous incarceration for the male population and both characteristics were not associated with IGRA positivity in females (Supplementary Table 3).

**Table 3:** Crude and adjusted odds ratios for IGRA positivity in people deprived of liberty evaluated in a tuberculosis cross-sectional survey, by gender (*n* = 884)

Variables	Male (*n* = 653)				Female (*n* = 231)			
	cOR (95% CI)	*p*-value	aOR (95% CI)	*p*-value	cOR (95% CI)	*p*-value	aOR (95% CI)	*p*-value
Age categories								
18–25 years old	Reference		Reference		Reference			
26–35 years old	0.94 (0.60–1.14)	0.800	0.69 (0.42–1.13)	0.140	1.88 (0.80–4.81)	0.200		
36–45 years old	0.98 (0.60–1.59)	> 0.900	0.79 (0.46–1.36)	0.400	1.69 (0.68–4.53)	0.300		
Above 46 years old	0.82 (0.45–1.52)	0.500	0.66 (0.34–1.30)	0.200	2.60 (0.93–7.64)	0.073		
Schooling								
Complete basic education or higher	Reference				Reference			
Incomplete basic education	1.50 (1.10–2.07)	0.012	1.46 (1.03–2.07)	0.034	0.90 (0.50–1.85)	0.700		
Race								
White	Reference				Reference			
Black/Mixed and Others	1.28 (0.87–1.87)	0.200			1.85 (0.84–4.53)	0.150		
Current incarceration time								
<1 year	Reference		Reference		Reference		Reference	
1–2 years	1.58 (1.07–2.36)	0.023	1.14 (0.71–1.83)	0.600	1.74 (0.87–3.46)	0.110	1.87 (0.91–3.82)	0.084
3–4 years	2.59 (1.57–4.36)	< 0.001	2.15 (1.18–3.92)	0.012	0.98 (0.30–2.77)	> 0.900	0.82 (0.23–2.49)	0.700
5 years or above	2.98 (1.90–4.47)	< 0.001	2.85 (1.66–4.90)	< 0.001	1.53 (0.63–3.57)	0.300	1.30 (0.50–3.19)	0.500
Previous incarceration	2.80 (1.94–4.06)	< 0.001	2.81 (1.85–4.25)	< 0.001	1.53 (0.86–2.76)	0.150	1.42 (0.77–2.64)	0.250
Previous contact with TB case in the same cell	1.49 (1.06–2.09)	0.021	1.13 (0.77–1.66)	0.500	1.17 (0.62–2.18)	> 0.900		
Previous TB history	3.94 (2.00–8.69)	< 0.001	2.99 (1.39–6.42)	0.005	5.20 (1.77–17.3)	0.004	5.93 (1.97–20.2)	0.002
Current tobacco use	1.17 (0.82–1.66)	0.400			0.97 (0.43–2.04)	> 0.900		
Alcohol abuse*	0.54 (0.28–1.02)	0.057			Not included*			
Drug use	0.85 (0.56–1.28)	0.400			0.70 (0.22–1.87)	0.500		

Abbreviations: *cOR* Crude Odds Ratio, *aOR* Adjusted Odds Ratio, *CI* Confidence Interval, *TB* Tuberculosis

*Alcohol abuse was only reported by one female participant and it was not included in the univariate analysis

Considering the 770 sputum samples available in seven prisons, we detected 20 individuals (2.6%, 20/770, 95% CI 1.6–3.9%) with positive Xpert results in our sample, corresponding to a point prevalence of 2597 cases per 100,000 PDL evaluated (95% CI 1687–3977). Three (15%, 3/20) of the positive Xpert tests also detected Rifampicin resistance (two samples from Manaus male prison and one sample from Venâncio Aires prison). Also, nine individuals had a Xpert trace result, these individuals were also referred to clinical evaluation by prison healthcare staff.

## Discussion

In this cross-sectional evaluation of eight Brazilian prisons, we found that over half of the individuals evaluated had a positive IGRA result and a high prevalence of active TB disease detected by Xpert. Male prisons had higher IGRA positivity than female prisons. The risk associated with IGRA positivity in male individuals were less education, previous incarceration, longer time of current incarceration and previous TB history; in female prisons, IGRA positivity was only associated with previous TB history.

Previous assessments utilizing tuberculin skin testing (TST) or IGRA in Brazilian prisons have shown TST/IGRA positivity between 11.7 and 73.0% [10, 13, 14, 22, 25–27], with the latest evaluation performed in 2021. To our knowledge, the Porto Velho male prison had a proportion of IGRA positivity (84.0%, 95% CI 75.3–90.1) above the highest rate previously recorded for Brazilian prisons. Although there is evidence that point TST or IGRA evaluation might be biased to assess the true prevalence of MTB infection due to false-positives and a significant proportion of individuals that do not convert despite high exposure to disease [28], these findings can be used to evaluate trends over time within the population and can still be used for estimates of the annual risk of infection of the population [17].

The prevalence of active TB disease detected by positive Xpert in our sample was also high, 2597 cases per 100,000 PDL, within the previously estimates for prevalence in South America region [4]. Similar discussions about bias introduced by false-positive Xpert results in surveys have been debated in the literature [29], however recent evidence has suggested that the Xpert specificity for similar screening studies is above 99% [30]. However, we did not collect information about the initial medical consultations, chest X-rays and culture results or symptom status for the individuals with positive Xpert results. It is important to reiterate that the burden of disease could be overestimated by false positives due to other reasons such as recently finalized TB treatment.

In context of previous evidence that carceral settings influence the national TB epidemic [6, 7], our findings reveal an urgent need for new strategies of TB control in prisons. We also evidence a significant heterogenicity in the IGRA positivity between prisons, suggesting different disease transmission profiles between these populations. New proposed approaches that might mitigate disease burden should account for these differences and could include biomedical interventions such as systematic active case finding efforts incorporating sputum and chest X-ray evaluation [31] or structural reforms of prison conditions and policy [7]. These efforts can not only reduce transmission by early identification and treatment of affected individuals, but also avoid the diagnostic delay of passive detection that could lead to post-TB lung disease and other complications [32].

Currently, national guidelines do not recommend tuberculosis preventive treatment (TPT) in prison settings [9]. Our findings of general high prevalence of IGRA positivity could be used to inform current discussions to update the recommended practices and reconsider including TPT as a strategy of disease control within the population. In some settings, considering that most individuals might already have a positive TST or IGRA result, it could be feasible to perform TPT in all individuals at risk without previous immunological testing, given that active disease is ruled out in those individuals eligible for TPT. These possible approaches have been shown to be well accepted and cost-effective in similar low and middle income countries [33]. Also, since the risk of developing active TB remains high years after incarceration [34], strategies to implement TPT prior to release or after incarceration could reduce the transmission burden to the overall population. However, implementation of such new strategies requires planning and assessment of feasibility with health authorities, accounting for heterogeneity between prisons, to promote highly efficient healthcare interventions in these vulnerable populations.

Our study also support earlier findings that incarceration time is strongly associated with TST/IGRA positivity [11, 14, 35]. This association was stronger for longer periods of exposure, despite previous reports that over a quarter of individuals can present TST conversion in a single year of follow-up [36]. Previous incarceration also indicates longer time at risk in this high burden environment. However, we did not encounter this association when evaluating the female prisons model, despite identifying a trend of increasing positivity over time of incarceration in females without previous prison and reports suggesting that the same is valid for both populations [14]. We hypothesize that this finding is due to the smaller number of females individuals evaluated and that in our sample incarceration time and previous incarceration proportions were significantly lower compared to the male individuals. Despite the absence of national estimates of this data stratified by gender, a survey performed in 12 prisons in a single state identified similar discrepancies in prison profiles of male and female units [14].

These findings should be evaluated in the light of the study limitations. We performed a random selection of individuals for inclusion not accounting for each facility characteristics. It is common for PDLs to be unevenly allocated in cell blocks due to crime profile or gang affiliations, that could lead to different crowding or demographics conditions distributed across the prison introducing bias, considering our small coverage of the total prison population. Moreover, except Venâncio Aires, all cities where the study was conducted are state capitals. We have no reason to believe that prison characteristics significantly differ within each state, however the TB incidence reported in those capitals is higher than the overall state [37]. Also, despite the randomization for individual selection, study sites were not selected randomly but chosen due to the presence of experienced research teams in prison healthcare, which could have influenced individual participation in procedures, previous site healthcare access and might not reflect the reality of other prison settings. Finally, the follow-up of the detected TB cases was performed by the prison healthcare units, so our report doesn’t include information about medical evaluation or further phenotypic and genotypic testing of the resistant TB cases.

## Conclusion

In conclusion, this multicenter survey demonstrates that tuberculosis infection and active disease remain highly prevalent in Brazilian carceral settings, with risk strongly associated with cumulative exposure to incarceration. These findings underscore the urgent need for systematic and comprehensive tuberculosis screening, prevention, and control strategies in prisons, including the potential evaluation of new preventative technologies such as new TB vaccines.

## Supplementary Information

Supplementary Material 1.

Supplementary Material 2.

## Data Availability

The dataset used for the present study are available upon reasonable request to the corresponding author.

## Abbreviations

IGRA: Interferon gamma release assay
TST: Tuberculin skin testing
TB: Tuberculosis
TPT: Tuberculosis Preventive Treatment
MTB: Mycobacterium tuberculosis
Xpert: GeneXpert Ultra
PDL: People deprived of liberty
