# Supplementary material for: Interferon gamma release assay and sputum GeneXpert positivity for tuberculosis burden detection in people deprived of liberty in Brazil: a cross-sectional study
Source: BMC Public Health. 2026 May 12;26:2053. doi: 10.1186/s12889-026-27643-3 (PMC13335293; doi:10.1186/s12889-026-27643-3)
Supplement: Supplementary file 1 — Supplementary Material 1. [file 12889_2026_27643_MOESM1_ESM.docx]

**SUPPLEMENTARY TABLES**

**Supplementary Table 1.** Characteristics of people deprived of liberty evaluated in a tuberculosis cross-sectional survey, by gender (n = 884).

|  |  | **Gender** | |  |
| --- | --- | --- | --- | --- |
| **Variables** | **Overall** N = 884*^1^* | **Male** N = 653*^1^* | **Female** N = 231*^1^* | **p-value***^2^* |
| **Age categories** |  |  |  | 0.400 |
| 18 – 25 years old | 154 (17.4%) | 114 (17.5%) | 40 (17.3%) |  |
| 26 – 35 years old | 389 (44.0%) | 295 (45.2%) | 94 (40.7%) |  |
| 36 – 45 years old | 238 (26.9%) | 174 (26.6%) | 64 (27.7%) |  |
| Above 46 years old | 103 (11.7%) | 70 (10.7%) | 33 (14.3%) |  |
| **Race** |  |  |  | 0.200 |
| White | 177 (20.0%) | 138 (21.1%) | 39 (16.9%) |  |
| Black/Mixed and Others | 707 (80.0%) | 515 (78.9%) | 192 (83.1%) |  |
| **Schooling** |  |  |  | <0.001 |
| Incomplete basic education | 425 (48.1%) | 340 (52.1%) | 85 (36.8%) |  |
| Complete basic education or higher | 459 (51.9%) | 313 (47.9%) | 146 (63.2%) |  |
| **Previously incarcerated** | 631 (71.4%) | 499 (76.4%) | 132 (57.1%) | <0.001 |
| **Previous contact with TB case in the same cell** | 291 (32.9%) | 230 (35.2%) | 61 (26.4%) | 0.014 |
| **Current incarceration time** |  |  |  | <0.001 |
| <1 year | 340 (38.5%) | 205 (31.4%) | 135 (58.4%) |  |
| 1-2 years | 247 (27.9%) | 198 (30.3%) | 49 (21.2%) |  |
| 3-4 years | 121 (13.7%) | 102 (15.6%) | 19 (8.2%) |  |
| 5 years or above | 176 (19.9%) | 148 (22.7%) | 28 (12.1%) |  |
| **Previous TB history** | 76 (8.6%) | 61 (9.3%) | 15 (6.5%) | 0.200 |
| **Positive IGRA result** | 474 (53.6%) | 404 (61.9%) | 70 (30.3%) | <0.001 |
| **Xpert results^3^** |  |  |  | <0.001 |
| Positive | 20/770 (2.5%) | 15/543 (2.7%) | 2/227 (0.8%) |  |
| Trace | 9/770 (1.1%) | 3/543 (0.5%) | 1/227 (0.4%) |  |
| Negative | 741/770 (96.4%) | 517/543 (96.8%) | 224/227 (98.8%) |  |
| Missing | 114 | 110 | 4 |  |
| **Living with HIV** | 13 (1.5%) | 8 (1.2%) | 5 (2.2%) | 0.300 |
| **Alcohol abuse** | 41 (4.6%) | 40 (6.1%) | 1 (0.4%) | <0.001 |
| **Current tobacco smoking** | 228 (25.8%) | 191 (29.2%) | 37 (16.0%) | <0.001 |
| **Illicit drug use** | 137 (15.5%) | 116 (17.8%) | 21 (9.1%) | 0.002 |
| *^1^*Median (Q1 - Q3); n (%). *^2^*Pearson's Chi-squared test; Fisher's exact test. ^3^Salvador site did not perform sputum testing and 7 other sputum samples were missing from the remaining prisons. Abbreviations: IGRA: interferon Gamma release assay; TB: Tuberculosis; Xpert: GeneXpert Ultra. | | | | |

**Supplementary Table 2.** Positivity of IGRA in people deprived of liberty evaluated in a tuberculosis cross-sectional survey, stratified by current incarceration time and previous incarceration history (n = 884)

|  |  | **IGRA Result - Male** | | | **IGRA Result - Female** | | |
| --- | --- | --- | --- | --- | --- | --- | --- |
| **Previously incarcerated** | **Current incarceration time** | **Negative**  N = 88*^1^* | **Positive**  N = 66*^1^* | **p-value***^2^* | **Negative**  N = 74*^1^* | **Positive**  N = 25*^1^* | **p-value^2^** |
| **No** | <1 year | 40 (76.9%) | 12 (23.1%) | <0.001 | 53 (79.1%) | 14 (20.9%) | 0.037 |
|  | 1-2 years | 21 (61.8%) | 13 (38.2%) |  | 11 (78.6%) | 3 (21.4%) |  |
|  | 3-4 years | 10 (45.5%) | 12 (54.5%) |  | 4 (66.7%) | 2 (33.3%) |  |
|  | 5 years or above | 17 (37.0%) | 29 (63.0%) |  | 6 (50.0%) | 6 (50.0%) |  |
| **Previously incarcerated** | **Current incarceration time** | **Negative**  N = 161*^1^* | **Positive**  N = 338*^1^* | **p-value***^2^* | **Negative**  N = 87*^1^* | **Positive**  N = 45*^1^* | **p-value***^3^* |
| **Yes** | <1 year | 64 (41.8%) | 89 (58.2%) | <0.001 | 46 (67.6%) | 22 (32.4%) | 0.574 |
|  | 1-2 years | 57 (34.8%) | 107 (65.2%) |  | 19 (54.3%) | 16 (45.7%) |  |
|  | 3-4 years | 19 (23.8%) | 61 (76.3%) |  | 10 (76.9%) | 3 (23.1%) |  |
|  | 5 years or above | 21 (20.6%) | 81 (79.4%) |  | 12 (75.0%) | 4 (25.0%) |  |
| *^1^*n (%) **Percentages described by row;** *^2^*Cochran-Armitage test for trend | | | | | | | |

**Supplementary Table 3.** Adjusted odds ratios for IGRA positivity in people deprived of liberty, sensitivity analysis of the original model performed excluding people with previous TB history, by gender (n=808).

|  | **Males (n = 592)** | | **Females (n = 216)** | |
| --- | --- | --- | --- | --- |
| **Variables** | **aOR (95% CI)** | **p-value** | **aOR (95% CI)** | **p-value** |
| **Age categories** |  |  |  |  |
| 18 – 25 years old | Reference |  |  |  |
| 26 – 35 years old | 0.69 (0.42 - 1.14) | 0.150 |  |  |
| 36 – 45 years old | 0.80 (0.46 - 1.38) | 0.400 |  |  |
| Above 46 years old | 0.64 (0.32 - 1.28) | 0.200 |  |  |
| **Schooling** |  |  |  |  |
| Complete basic education or higher | Reference |  |  |  |
| Incomplete basic education | 1.57 (1.09 - 2.25) | 0.015 |  |  |
| **Current incarceration time** |  |  |  |  |
| <1 year | Reference |  | Reference |  |
| 1-2 years | 1.20 (0.74 - 1.94) | 0.500 | 1.62 (0.80 – 3.25) | 0.200 |
| 3-4 years | 2.25 (1.22 - 4.16) | 0.009 | 0.91 (0.27 – 2.61) | 0.900 |
| 5 years or above | 2.81 (1.60 - 4.91) | <0.001 | 1.48 (0.60 – 3.49) | 0.400 |
| **Previous incarceration** | 2.86 (1.87 - 4.38) | <0.001 | 1.44 (0.80 – 2.63) | 0.200 |
| **Previous contact with TB case in the same cell** | 1.20 (0.80 – 1.79) | 0.400 |  |  |
| Abbreviations: aOR: Adjusted Odds Ratio; CI: Confidence Interval; TB: Tuberculosis. | | | | |
