# Supplementary material for: Interferon gamma release assay and sputum GeneXpert positivity for tuberculosis burden detection in people deprived of liberty in Brazil: a cross-sectional study
Source: BMC Public Health. 2026 May 12;26:2053. doi: 10.1186/s12889-026-27643-3 (PMC13335293; doi:10.1186/s12889-026-27643-3)
Supplement: Supplementary file 2 — Supplementary Material 2. [file 12889_2026_27643_MOESM2_ESM.docx]

**SUPPLEMENTARY MATERIAL – ANNEX 1**

**SOCIODEMOGRAPHIC QUESTIONNAIRE**

**MANUSCRIP TITLE: Interferon gamma release assay and sputum GeneXpert positivity for tuberculosis burden detection in people deprived of liberty in Brazil: a cross-sectional study.**

| **1. Identification** |
| --- |

| Prison Unit: |  | Cell ward: |  | Cell block: |  | Cell number: |  |
| --- | --- | --- | --- | --- | --- | --- | --- |

| 1.1 Name: |  | | | | | | | |
| --- | --- | --- | --- | --- | --- | --- | --- | --- |
| 1.2 Mother name: | |  | | | | | | |
| 1.3 Date of birth: | | |  | / |  | / |  |  |

1.4 Sex:

⚪ Male

⚪ Femamle

1.5 Race:

⚪ White

⚪ Black

⚪ Mixed

⚪ Yellow

⚪ Indigenous

⚪ No declaration

| 1.6 RGI (prison ID): |  | |
| --- | --- | --- |
| 1.7 City/State of residence before prison: | |  |

| **2. Sociodemographic data** |
| --- |

2.1 Marital Status:

⚪ Single

⚪ Married

⚪ Widowed

⚪ Divorced

⚪ Stable union

2.2 Schooling

| ⚪ No schooling  ⚪ Incomplete first 4 years of basic education  ⚪ Complete first 4 years of basic education  ⚪ Incomplete first 9 years of basic education  ⚪ Complete first 9 years of basic education | ⚪ Incomplete high school  ⚪ Complete high school  ⚪ Incomplete higher education  ⚪ Complete higher education  ⚪ Post-graduation |
| --- | --- |

2.3 Family income:

⚪ Less than 1 minimum wage (< R$ 1.518)

⚪ 1 to 2 minimum wages (R$ 1.518 to 3.036)

⚪ 3 to 4 minimum wages (R$ 4.554 to 6.072)

⚪ More than 4 minimum wages (> R$ 6.072)

⚪ Not informed

| **3. Comorbidities** |
| --- |

| ⬜ Undernutrition  ⬜ Diabetes mellitus type 1  ⬜ Diabetes mellitus type 2  ⬜ HIV infection  ⬜ Current smoking  ⬜ Alcohol abuse disorder | ⬜ Illicit drug use  ⬜ Previous tuberculosis (TB)  ⬜ Kidney disease  ⬜ Haematological disease  ⬜ Liver disease  ⬜ Obesity | ⬜ Immunodeficiency  ⬜ Neurological disease  ⬜ Lung disease  ⬜ Asthma  ⬜ Other comorbidities, specify:  ___________________________ |
| --- | --- | --- |

| **4. Data from the investigation** |
| --- |

4.1. Time of current incarceration

| ⚪ 1 year  ⚪ 2 years  ⚪ 3 years  ⚪ 4 years | ⚪ 5 years  ⚪ 6 a 9 years  ⚪ 10 years or more |  |  |
| --- | --- | --- | --- |

4.2 Was there any change of prison unit in the current incarceration?

⚪Yes, how many prison units:_________________________________________________________________

⚪ No

4.3. Have you ever been incarcerated before?:

⚪ Yes

⚪ No

4.4 Receives visits in the prison:

⚪ Yes, what frequency: _____________________________________________________________________

⚪ No

If yes, by whom?

| ⬜ Father / Stepfather  ⬜ Mother / Stepmother  ⬜ Wife/Husband | ⬜ Boyfriend/Girlfriend  ⬜ Sister/Brother  ⬜ Son/Daughter | ⬜ Friends  ⬜ Lawer  ⬜ Others________________________________________ |
| --- | --- | --- |

4.5 Have you ever had any cellmates with TB in your cell?

⚪ Yes

⚪ No

⚪ Ignored

4.6 Are you currently on TB treatment?

⚪ Yes

⚪ No

4.7 Have you ever been treated for TB before?

⚪ Yes, in the year:____________________________________________________________________

⚪ No

4.8. Do you use any medication?

⚪ Yes, which one?___________________________________________________________________________

⚪ No
